# Spatial accessibility and equity of traditional Chinese medicine hospitals in Eastern China: application of improved two-step floating catchment area method

**DOI:** 10.3389/fpubh.2026.1741264

**Published:** 2026-02-05

**Authors:** Caiming Xu, Tingyu Mu, Yulian Liu, Rixiang Xu

**Affiliations:** 1 School of Law, Hangzhou City University, Hangzhou, Zhejiang, China; 2 School of Nursing, Anhui Medical University, Hefei, China; 3 Ningbo Municipal Hospital of Traditional Chinese Medicine, Ningbo, China; 4 School of Medical Humanities, Anhui Medical University, Hefei, China

**Keywords:** equity, health resources, public hospital, spatial accessibility, traditional Chinese medicine

## Abstract

**Purpose:**

Despite the growing role of Traditional Chinese Medicine (TCM) in China, little is known about the spatial accessibility and equity of TCM hospital resources at high geographic resolution. This study quantifies the spatial accessibility (SA) of TCM hospitals and assess the equity in resource distribution across Zhejiang Province to identify distributional disparities and inform policy responses.

**Methods:**

We applied a Two-Step Floating Catchment Area (2SFCA) framework augmented with navigation-derived travel times from a real-time route API to estimate SA for 99 public TCM hospitals and 25,601 residential demand points; equity was evaluated using Gini coefficients for four core resources (TCM physicians, TCM pharmacists, inpatient beds, and high-value TCM equipment), and spatial clustering was examined via Moran's I.

**Results:**

Among the 25,601 residential points analyzed, 93.47% were within a 60 min driving distance of a TCM hospital, covering 97.21% of the provincial population. However, accessibility varied considerably by municipality: Hangzhou exhibited the highest SA scores, while Lishui demonstrated consistently poor equity across all resource indicators. Gini coefficients ranged from 0.365 for TCM pharmacists (indicating proper equity) to 0.505 for medical equipment (indicating high inequity). Spatial autocorrelation analysis revealed distinct clustering patterns, with accessibility shaped by both topographical constraints and disparities in resource allocation.

**Conclusion:**

While Zhejiang achieves high overall SA to TCM hospitals, substantial inequities in resource allocation remain. Policy implications include targeted redistribution of high-value assets (for example, regional equipment-sharing consortia), deployment of mobile diagnostic and treatment units for remote and insular areas, incentives to attract TCM practitioners to underserved localities, and strengthening county-level service capacity to reduce intra-municipal disparities. These measures would help translate broad coverage into more equitable access to TCM services.

## 1 Introduction

Ensuring equitable access to healthcare is a pressing global challenge, and many countries are recognizing the value of integrating traditional medicine with conventional care. The World Health Organization's Traditional Medicine Strategy (2025–2034) explicitly envisions universal access to traditional, complementary, and integrative medicine (TCIM) as part of primary healthcare (1). In China, Traditional Chinese Medicine (TCM) constitutes a fundamental component of the national health system (2, 3), supported by longstanding policies aimed at its coordinated development with conventional medicine since the 2009 health reform (4). For example, by 2020 China counted over 4,000 of public TCM hospitals and sizable outpatient volumes at these institutions (5). Public TCM hospitals serve as the institutional backbone of this system, critical for delivering and preserving TCM services. Consequently, promoting equity in access to public TCM hospitals has become a central objective of recent healthcare reforms (6).

Despite policy emphasis, macro-level analyses indicate persistent regional disparities in the distribution of TCM resources across China, suggesting underlying inequities in spatial access (7). Spatial accessibility (SA) is a key determinant of health equity and defined as the relative ease with which individuals can physically reach healthcare facilities (8). Limited SA can exacerbate health disparities, particularly for vulnerable groups who may forgo or delay necessary care. While GIS-based SA assessments have been extensively applied to general healthcare facilities such as hospitals and clinics (9–11), they have rarely focused on TCM-specific providers. This gap is significant because TCM utilization patterns differ notably from those of Western medicine. TCM services are often sought for chronic disease management, rehabilitation, and preventive care, rather than acute emergencies (12, 13). Patient behavior, including willingness to travel and preference for traditional modalities, may therefore follow distinct spatial patterns, necessitating a dedicated and tailored accessibility analysis. To address this gap, this study conducts a high-resolution spatial assessment of SA and equity for public TCM hospitals in Zhejiang Province, China. Zhejiang represents an ideal case for several reasons: it is an economically dynamic province with pronounced topographical diversity (from coastal plains to mountainous regions), leading to varied infrastructure development and population distribution. More importantly, Zhejiang has been designated both as a national pilot zone for comprehensive TCM reform and for the “common prosperity” initiative aimed at reducing regional disparities, making it a critical testing ground for evaluating and informing equitable health resource policies.

Methodologically, improving the precision of accessibility measurement is essential to close this gap. Network-based travel distances or Euclidean metrics can distort travel-time costs, particularly in regions with complex topography or heterogeneous traffic conditions (14, 15). To enhance realism, this study integrates navigation-derived travel times obtained from a real-time route API with a Two-Step Floating Catchment Area (2SFCA) framework (14), applies a Gaussian decay function to model distance-sensitive utilization (16, 17), and combines high-resolution demand points with resource-specific supply indicators. We further assess equity using Gini coefficients (18) and characterize spatial clustering with Global and Local Moran's I (19). The integration of real-time navigation data, fine-grained population locations, and resource-level equity metrics represents a methodological advance over conventional accessibility studies and is particularly suited to capturing the spatial logic of TCM service use.

Guided by the foregoing rationale, this paper advances three explicit objectives. First, it quantifies spatial accessibility to public TCM hospitals across Zhejiang at village/community resolution using a 2SFCA approach augmented by navigation-derived travel times. Second, it evaluates the equity of four core TCM resource types (TCM physicians, registered TCM pharmacists, inpatient beds, and high-value TCM equipment) using Gini coefficients and maps the spatial clustering of accessibility scores with Global and Local Moran's I. Third, it interprets these spatial patterns to derive practical, evidence-based policy recommendations. By treating public TCM hospitals as a distinct supply category and combining real-time travel data with high-resolution demand information and equity metrics, the study seeks to contribute both methodological insight and actionable guidance for TCM resource planning.

## 2 Materials and methods

### 2.1 Study area

Zhejiang Province, situated along the eastern coast of China, is an economically advanced region with a population of approximately 65 million. As of the end of 2021, the province was administratively divided into 11 prefecture-level cities, 90 counties (or districts), 1,364 towns (or subdistricts), and 25,601 villages (or communities). Geo-graphically, Zhejiang exhibits a southwest-to-northeast topographical gradient: low-lying alluvial plains dominate the northeastern region, hills and coastal plains de-fine the eastern area, the central zone comprises hills and basins, and the southwestern part is largely mountainous. This complex and varied terrain shapes both population distribution and infrastructure accessibility, making the province a compelling case for spatial health equity research.

Recognizing its representative value, Zhejiang was selected in 2021 as one of the initial pilot provinces for comprehensive TCM reform, and uniquely, as the sole pilot zone for the national “common prosperity” initiative (a national policy aimed at reducing regional and socioeconomic disparities). These dual designations underscore Zhejiang's strategic role in advancing healthcare system reform, particularly in integrating TCM into mainstream service delivery. However, despite strong urban healthcare infrastructure, rural and mountainous areas within the province face persistent barriers to accessing essential services, including TCM. Such geographic disparities raise important questions regarding the equity of resource distribution and the adequacy of current policy responses in addressing spatial and socioeconomic variation in healthcare access.

### 2.2 Study data

#### 2.2.1 Point-of-supply data

**Figure 1:**
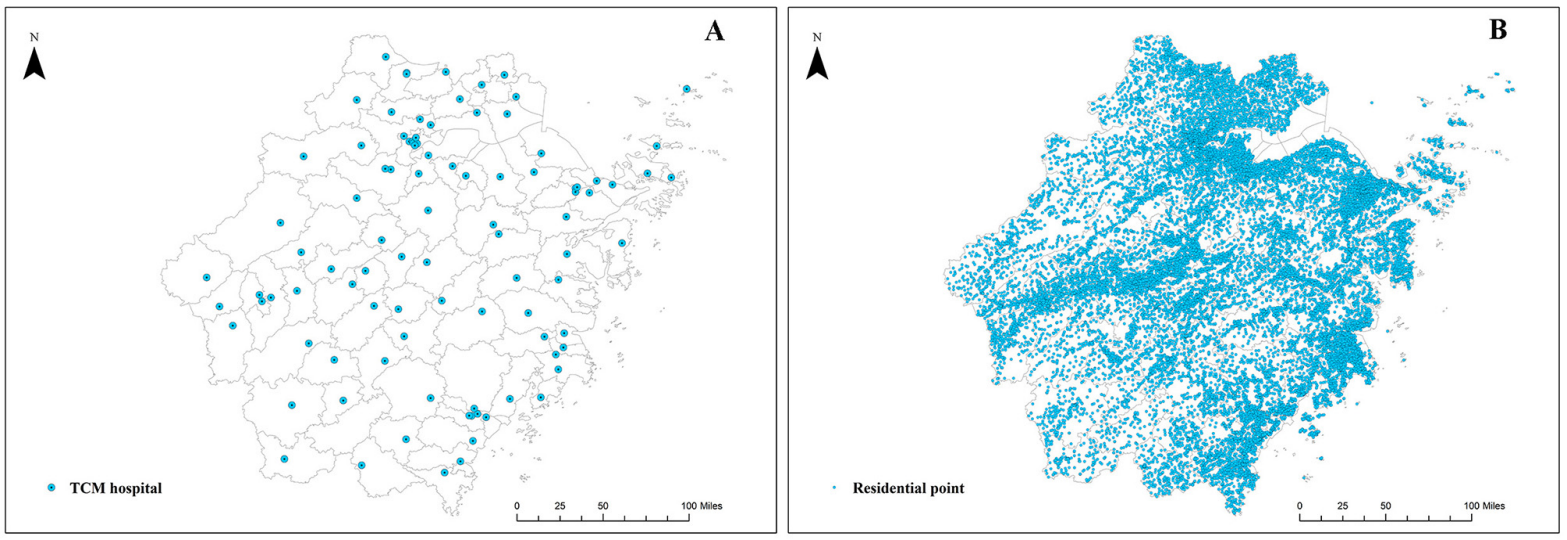
The spatial distribution of public traditional Chinese medicine hospitals **(A)** and residential points **(B)**.

We conducted a cluster sampling of all 99 TCM hospitals in Zhejiang Province during 2021 to ensure full coverage and representativeness. Key variables gathered included the number of licensed TCM physicians, registered TCM pharmacists, inpatient beds, and high-value TCM medical equipment. We define “high-value equipment” as diagnostic and therapeutic devices whose unit cost exceeds RMB 5,000, in accordance with provincial statistical guidelines. This threshold excludes lower-value items such as acupuncture needles, gua sha tools, and basic cupping sets. Examples of included equipment comprise TCM pulse diagnosis instruments, electroacupuncture generators, decoction machines, and advanced herbal extraction units; quantities of each device were tallied per hospital. These four indicators were selected because they are widely recognized in Chinese health policy documents and national evaluation frameworks as core components of hospital TCM service capacity. These data were sourced from the provincial administrative authority responsible for TCM governance in Zhejiang, ensuring consistency and institutional validity. Hospital classification and geographic coordinates were also obtained. In the subsequent spatial accessibility calculation, the supply capacity for each resource type was analyzed separately. That is, four independent 2SFCA analyses were conducted, each using one of the four resource indicators as the sole measure of supply capacity. This approach allows us to examine accessibility patterns specific to each resource category. A summary of institutional characteristics and their spatial distribution is provided in Supplementary Table 1 and Figure 1A, respectively.

#### 2.2.2 Point-of-demand data

This study identified village committees and community committees across Zhejiang Province as the demand points for SA analysis. These units represent the smallest administrative divisions in China's urban and rural governance structures, typically characterized by localized self-management. Usually located near the central areas of their respective villages or communities, these administrative bodies serve as focal points for public service delivery. Their spatial distribution closely reflects the population's geographic settlement pattern, making them suitable proxies for approximating residents' access to healthcare facilities.

A total of 25,601 demand points in Zhejiang province were identified based on official data published by the National Bureau of Statistics. Geographic coordinates for each point were subsequently retrieved in bulk using the AutoNavi Map (Amap) open platform Application Programming Interface (API) (Figure 1B). Quality control procedures included: removal of duplicate entries based on administrative codes; verification of coordinate plausibility by checking for points falling outside Zhejiang's provincial boundary; and cross-referencing with official gazetteers to correct obvious geolocation errors. Population data for 2021 were sourced from the WorldPop database (100 m resolution). The population count was assigned to each demand point based on the grid cell in which it was located. For the very few points where WorldPop data were missing, the population was imputed using the average value of adjacent points within the same township.

#### 2.2.3 Travel time data

The acquisition of travel time costs from origin (residential locations) to destination (sampled hospitals), known as the Origin-Destination (OD) cost matrix, is a critical step in calculating SA. In this study, the OD cost matrix was generated using the driving route API provided by the Amap Open Platform. The specific procedure and implementation details were as follows: Data processing and API calls were executed in Python 3.11, utilizing the requests library for HTTP queries and the csv library for file I/O. The core API request followed this structure: https://restapi.amap.com/v3/direction/driving?key={your_key}&origin={start_lon},{start_lat}&destination={end_lon},{end_lat}&strategy=0.

The critical parameter strategy=0 specifies the fastest route strategy. This method accounts for real-time traffic conditions, traffic signals, road quality, and other relevant factors, thereby offering a more accurate estimation of the time costs that residents incur when traveling to healthcare facilities. Residential areas were designated as the origins, while each sampled TCM hospital was considered the destination. Travel time data between these points were obtained in batches by writing custom code in a Python environment, interfacing with the Amap API. To ensure the accuracy and realism of the data, travel times were measured during both peak and off-peak traffic periods, with the average value used for subsequent analysis. Data validation and cleaning were integral to the workflow: API responses were checked for the infocode=0 (success) status; failed requests were logged and re-attempted after a delay. Returned duration values (in seconds) were converted to minutes. Outliers were filtered by removing any OD pairs with a travel time exceeding 1,440 min (24 h), as such durations are not representative of intra-provincial healthcare-seeking trips. Finally, the cleaned OD matrix was structured with columns: origin_id, destination_id, travel_time_min.

### 2.3 Research methods

#### 2.3.1 Two-step floating catchment area method

The conceptual foundation of the Two-Step Floating Catchment Area (2SFCA) method was initially explored by Radike, an American scholar, in the context of spatial accessibility measurement (20). This approach was later refined and formalized by Wang and Luo, who established the canonical 2SFCA methodology (21). The 2SFCA method involves two key steps for calculating accessibility. The first step focuses on the supply points, within which demand points are identified, and the supply-demand ratio is calculated to assess the level of service provision at each supply point. The second step aggregates these supply-demand ratios across multiple supply points to compute overall accessibility at each demand point. For more detailed methodological explanations, readers are referred to previous studies (22, 23).

The calculation process for the 2SFCA method is outlined in two formulas. Equation 1 calculates the supply-demand ratio for each supply point, while Equation 2 computes the accessibility for a specific demand point. To better reflect the gradual decrease in service utilization with increased travel time, we applied a Gaussian decay function to weight travel impedance. This choice was informed by its growing adoption in recent health accessibility research and its theoretical advantage of providing a continuous and smooth decay curve. Compared with inverse power or kernel density functions, the Gaussian function better captures travel behavior by avoiding sharp threshold effects. This approach has been successfully applied in recent empirical studies (24, 25). The weighted calculation is represented by Equation 3. Thus, the effective accessibility experienced by a demand point is the product of the hospital-level accessibility component and the distance-decay weight *G*(*d*_*ij*_).


$$\begin{array}{l} R_{j}=\frac{S_{j}}{\sum_{k\in \left\{d_{ij}\leq d_{0}\right\}D_{i}}} \end{array}$$ (1)

$$\begin{array}{l} A_{i}=\sum_{j\in \left\{d_{ij}\leq d_{0}\right\}}R_{j} \end{array}$$ (2)

$$\begin{array}{c} G\left(d_{ij}\right)=\{\begin{array}{c} \frac{e^{-\frac{1}{2}\times {\left(\frac{d_{ij}}{d_{0}}\right)}^{2}}-e^{-\frac{1}{2}}}{1-e^{-\frac{1}{2}}}\\ 0,d_{ij}\geq d_{0} \end{array}\;,\;d_{ij}\leq d_{0} \end{array}$$ (3)

Where *R*_*j*_ is the supply-demand ratio at supply point *j, S*_*j*_represents the supply capacity at point *j*, and *D*_*i*_ represents the demand intensity of demand point *i*. *A*_*i*_ is the SA of demand point *i. d*_*ij*_ is the travel time from demand point *i* to supply point *j. d*_0_ is the maximum travel time (threshold) for residents to drive to the supply point. *G*(*d*_ij_) represents the distance-based weighting coefficient applied to the interaction between demand point *i*and supply point *j*. We set the travel time thresholds (*d*_0_) at 60 min for county-level and 90 min for municipal-level TCM hospitals, aligning with the administrative hierarchy of healthcare services. This choice draws on Yue Jingkai's study (26), which employed similar thresholds in a navigation-based accessibility analysis of secondary and tertiary hospitals. Given the comparable classification, this serves as a justified and contextually relevant reference.

#### 2.3.2 Equity assessment

To evaluate the disparities in SA of TCM hospitals across different demand points in the sample area, we used the Gini coefficient to analyze the results (27). The Gini coefficient is a commonly used measure of inequality, where values below 0.2 indicate absolute equity, values between 0.2 and 0.3 represent relative equity, values between 0.3 and 0.4 denote reasonable equity, values between 0.4 and 0.5 signify significant disparity, and values above 0.5 indicate high inequity. The Gini coefficient is calculated using the following Equation 4.


$$\begin{array}{c} Gc=\frac{\sum_{ij}\left|A_{j}-A_{i}\right|}{2n^{2}A} \end{array}$$ (4)

where *Gc* is Gini coefficient; *A*_*i*_ and *A*_*j*_ are the *SA* of any two demand point *i* and *j*, respectively; *A* is the average *SA* in the Zhejiang province; and n is the total number of demand point.

#### 2.3.3 Spatial autocorrelation analysis

To identify and quantify spatial clustering patterns (spatial autocorrelation) and local hotspots/coldspots (spatial heterogeneity) in the spatial accessibility (SA) scores for the four core TCM resources across the 25,601 residential demand points, we employed both Global and Local Moran's I statistics. The spatial weights matrix was constructed and all spatial autocorrelation analyses were performed using ArcGIS (version 10.3).

(1) Global spatial autocorrelation (Global Moran's I)

This statistic measures the overall degree of spatial clustering or dispersion of the SA values across the entire study area. It indicates whether similar values tend to be located near each other (positive autocorrelation, clustering) or dissimilar values are near each other (negative autocorrelation, dispersion), or if the pattern is random. The spatial weights matrix was constructed using an inverse-distance weighting scheme with a distance threshold set to ensure each demand point had at least one neighbor. This choice was made because SA is expected to exhibit distance-dependent spatial dependency, and inverse-distance weighting better captures continuous spatial processes compared to contiguity-based (queen/rook) matrices. The Global Moran's I statistic is calculated as follows:


$$Moran^{'}s\;I=\frac{n\sum_{i=1}^{n}\sum_{j=1}^{n}W_{ij}\left(Y_{i}-\bar{Y}\right)\left(Y_{j}-\bar{Y}\right)}{\sum_{i=1}^{n}\sum_{j=1}^{n}w_{ij}\sum_{i=1}^{n}{\left(Y_{i}-\bar{Y}\right)}^{2}}$$ (5)

Where *n* represents the total number of objects of study within the research area, *W*_*ij*_ denotes the spatial weight value in the selected spatial weights matrix, *Y*_*i*_ and *Y*_*j*_ are the values of the study variable for the i-th and j-th objects of study in the research area, *Y* is the mean value of all objects of study within the spatial scope of the research. The value of Moran's I ranges approximately from −1 (perfect dispersion) to +1 (perfect clustering). A value near 0 suggests a random spatial pattern. The statistical significance of Moran's I was tested using a permutation approach (999 permutations) to generate a pseudo *p*-value, allowing us to reject the null hypothesis of spatial randomness (*p* < 0.05).

(2) Local spatial autocorrelation (Local Moran's *I*/LISA)

While Global Moran's I provides an overall measure, Local Moran's I (often referred to as LISA) identifies specific locations (demand points) and their neighborhoods where spatial clustering of similar values (high-high or low-low) or spatial outliers (high-low or low-high) is statistically significant. This allows for the mapping of local hotspots (clusters of high SA) and coldspots (clusters of low SA), as well as spatial anomalies. The Local Moran's I statistic for demand point *i* is calculated as:


$$\begin{array}{l} I_{i}=\frac{\left(x_{i}-\bar{x}\right)}{L^{2}}\overset{n}{\underset{j\neq i}{\sum }}W_{ij}\left(x_{j}-\bar{x}\right) \end{array}$$ (6)

Where *I*_*i*_ represents the Local Moran's *I* calculated for the *i*-th object of study, *x* denotes the mean value across all objects of study within the research area, *L*^2^ is the variance, n is the total number of objects of study, and *W*_*ij*_ corresponds to the spatial weight value within the chosen spatial weights matrix. *I*_*i*_ were categorized into four types based on the value at point *i (A*_*i*_*)* and the spatial lag (weighted average of neighbors):

High-High (HH): high SA at *i* surrounded by high SA neighbors (Hotspot).

Low-Low (LL): low SA at *i* surrounded by low SA neighbors (Coldspot).

High-Low (HL): high SA at *i* surrounded by low SA neighbors (High Outlier).

Low-High (LH): low SA at *i* surrounded by high SA neighbors (Low Outlier).

## 3 Results

### 3.1 Study characteristics

A total of 99 eligible TCM hospitals in Zhejiang Province were included in this analysis. Of these, 50.5% were classified as secondary hospitals, 83.8% as general TCM hospitals, and 82.8% as county- or district-level institutions. In terms of resource allocation, tertiary hospitals, general TCM hospitals, and county-level hospitals each accounted for over half of the province's aggregate supply of TCM physicians, pharmacists, inpatient beds, and high-value medical equipment. Detailed characteristics of the sampled hospitals are provided in Table 1. For the demand side, 25,601 village and community committees were used as proxy demand points. These points are predominantly clustered in the southeastern coastal corridor, the eastern littoral zones, and the Jinhua–Quzhou Basin, closely mirroring the province's population distribution. The geographic concentration of these residential points and their associated populations is illustrated in Figure 1.

**Table 1:** Characteristics of healthcare resources in public TCM hospitals in Zhejiang Province.

**Classification**	**Institution (*n* = 99)**	**TCM physician (*n* = 9, 698)**	**TCM pharmacist (*n* = 2,089)**	**Bed (*n* = 41,060)**	TCM medical equipment^*^ (*n* = 9,070)
**Hospitals level**					
Undetermined	14 (14.1)	301 (3.1)	87 (4.2)	1342 (3.3)	376 (4.1)
Second-level	50 (50.5)	3,246 (33.4)	871 (41.7)	14,077 (34.3)	3,318 (36.6)
Third-level	35 (35.4)	6,151 (63.4)	1,131 (54.1)	25,641 (62.5)	5,376 (59.3)
**Hospital type**					
Traditional Chinese medicine (general) hospital	82 (83.8)	8,514 (87.8)	1,858 (88.9)	33,490 (81.6)	7,554 (83.4)
Integrated Chinese and Western medicine hospital	12 (11.1)	938 (9.7)	189 (9.0)	6,379 (15.5)	1,068 (12.1)
Other TCM specialty hospitals	2 (2.0)	57 (0.6)	13 (0.6)	405 (1.0)	42 (0.5)
Orthopedic hospitals	3 (3.0)	189 (1.9)	29 (1.4)	786 (1.9)	367 (4.0)
**Administrative management**					
Provincial level	4 (4.0)	1,508 (15.5)	210 (10.1)	6,118 (14.9)	1,061 (11.7)
Municipal level	13 (13.1)	2,381 (24.6)	522 (25.0)	9,865 (24.0)	2,577 (28.4)
County (district) level	82 (82.8)	5,809 (59.9)	1,357 (65)	25,077 (61.1)	5,432 (59.9)

^*^TCM medical equipment worth more than 5000RMB.

### 3.2 Coverage area

**Figure 2:**
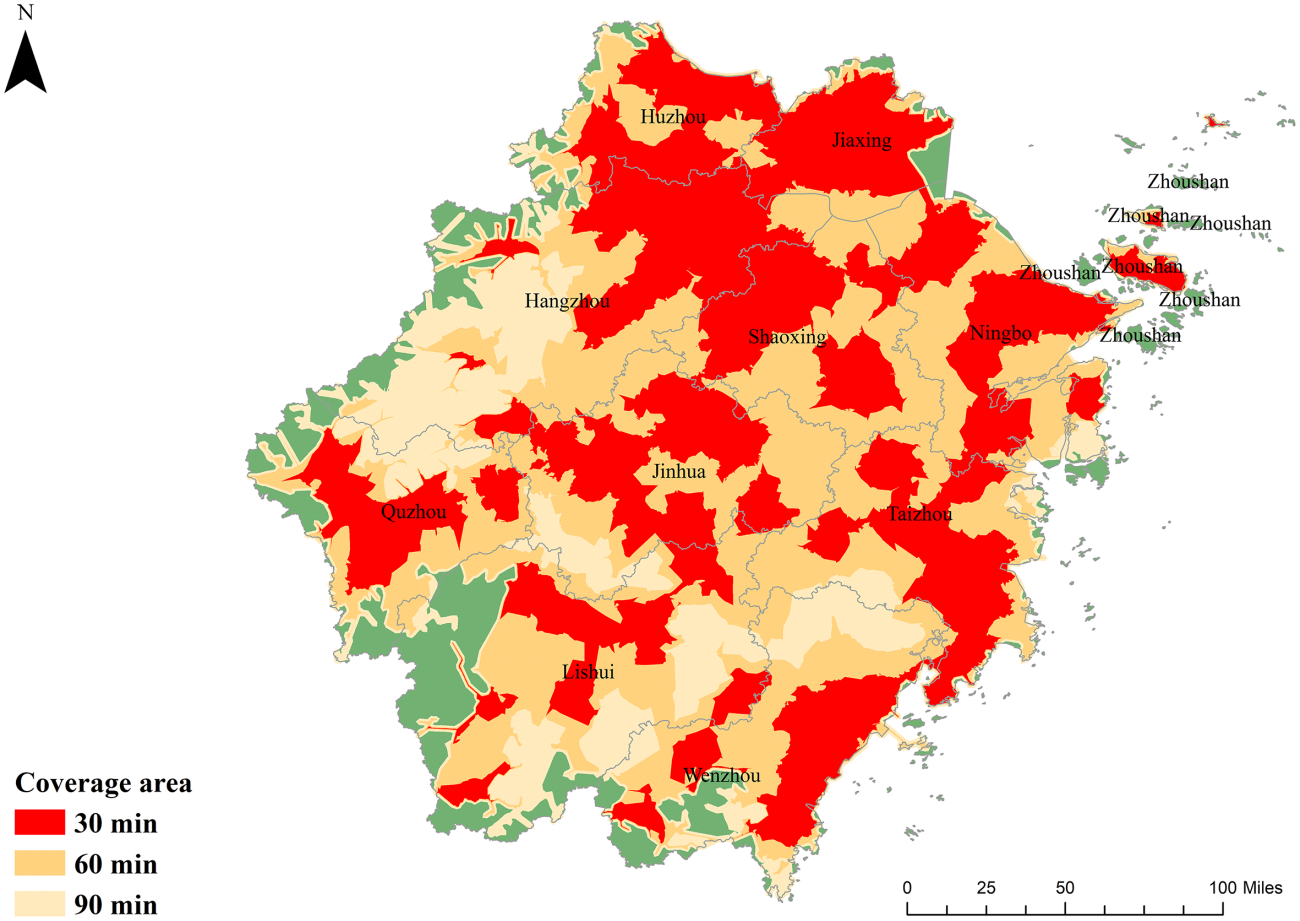
Coverage areas at different travel times for public TCM hospitals. *TCM medical equipment worth more than 5000RMB.

The spatial coverage of sampled TCM hospitals under varying travel-time thresholds is summarized in Table 2. Province-wide, 56.11% of residential points lie within a 30-min drive of at least one hospital, rising to 93.47% and 98.68% at the 60- and 90-min thresholds, respectively. When weighted by population, coverage increases to 71.45%, 97.21%, and 99.53% at the same intervals. This discrepancy between point-based and population-weighted metrics underscores that more densely inhabited areas enjoy disproportionately better access. Municipality level analysis reveals that, at a 30-min threshold, over half of all residential points in every city (except Lishui and Wenzhou) are served by a nearby TCM hospital. Increasing the threshold to 60 min brings coverage above 90% in all but Lishui, Wenzhou, and Zhoushan; notably, Jiaxing attains full point coverage. At 90 min, all municipalities except Zhoushan exceed 95% coverage, with Huzhou, Jiaxing, and Shaoxing achieving complete coverage. Across all thresholds, population-weighted coverage equals or surpasses point coverage, signaling that resource distribution favors population centers. Figure 2 maps these service areas. Most of Zhejiang falls within a 60-min catchment; the residual gaps at 90 min are confined to western border regions and the outlying islands of Zhoushan. These spatial patterns highlight the need for targeted interventions in peripheral and insular zones to close remaining accessibility gaps.

**Table 2:** Coverage of demand points and population by travel time to public TCM hospitals in Zhejiang province municipalities.

**Regional name**	**Coverage of demand points (%)**			**Coverage of population (%)**		
	**30 min**	**60 min**	**90 min**	**30 min**	**60 min**	**90 min**
Hangzhou	65.33	94.14	99.66	82.56	98.56	99.94
Huzhou	59.97	99.84	100.00	67.45	99.91	100.00
Jiaxing	67.84	100.00	100.00	71.24	100.00	100.00
Jinhua	54.69	94.88	99.81	70.74	98.47	99.96
Lishui	41.16	78.65	92.11	63.59	88.84	97.06
Ningbo	62.31	96.59	99.66	74.76	98.96	99.90
Quzhou	66.21	97.26	99.5	73.48	98.39	99.83
Shaoxing	63.11	98.86	100.00	74.69	99.54	100.00
Taizhou	58.24	97.30	99.65	73.95	99.28	99.92
Wenzhou	35.61	85.97	98.43	56.93	91.57	99.28
Zhoushan	65.29	79.12	82.52	75.13	86.90	89.03
The entire province	56.11	93.47	98.68	71.45	97.21	99.53

### 3.3 Spatial accessibility analysis

**Figure 3:**
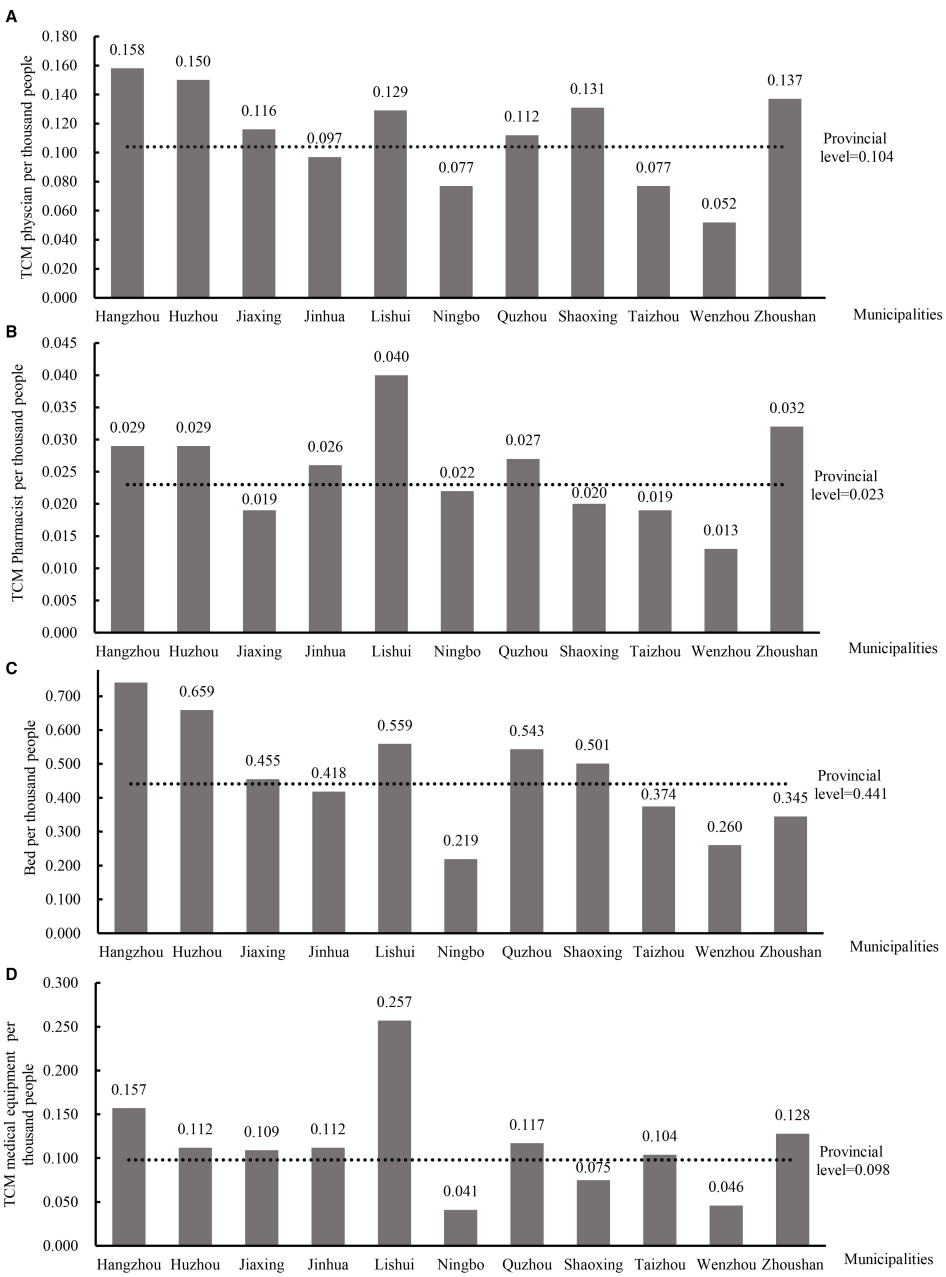
Spatial accessibility scores per 1,000 people for four core resources across the 11 municipalities in Zhejiang Province. **(A)** TCM physician; **(B)** TCM pharmacist; **(C)** bed; **(D)** TCM medical equipment.

Figure 3 presents municipal-level SA scores for four core TCM resource categories in Zhejiang Province. The provincial means for physician, pharmacist, bed, and equipment access were 0.104, 0.023, 0.441, and 0.098, respectively. At the upper end of the distribution, Hangzhou led physician accessibility (0.158), while Lishui topped pharmacist (0.040) and equipment (0.257) access; Hangzhou also achieved the highest bed score (0.740). Conversely, Wenzhou consistently ranked lowest for both physician (0.052) and pharmacist (0.013) SA, and Ningbo recorded the poorest bed (0.219) and equipment (0.041) accessibility. Several cities (including Jinhua, Ningbo, Taizhou, and Wenzhou) fell below the provincial average for physician access, while Jiaxing, Ningbo, Shaoxing, Taizhou, and Wenzhou underperformed on pharmacist SA. For bed access, Jinhua, Ningbo, Taizhou, Wenzhou, and Zhoushan scored below the mean, and equipment SA was similarly constrained in Ningbo, Shaoxing, and Wenzhou. These pronounced intermunicipal disparities underscore the need for targeted redistribution of TCM resources to bolster equity across the province. These pronounced disparities reflect significant spatial unevenness in TCM resource allocation, particularly in economically diverse and topographically complex regions. For instance, despite Lishui's relatively weaker economic status, it scores highly on pharmacist and equipment accessibility, likely due to the concentration of high-quality resources within a few key hospitals, which elevates overall average accessibility but may mask internal inequities.

### 3.4 Equity and spatial heterogeneity analysis

Table 3 summarizes municipal-level Gini coefficients for four categories of TCM resources in Zhejiang Province. At the provincial scale, the distribution of physicians (Gini = 0.382) and pharmacists (Gini = 0.365) falls within a reasonable equity; inpatient beds (Gini = 0.400) exhibit clear disparity; and medical equipment (Gini = 0.505) shows pronounced inequity. Notably, the inequity observed for capital-intensive resources such as medical equipment substantially exceeds that for human resources. This contrast highlights the need for differentiated policy strategies that address the unique allocation dynamics of physical assets vs. personnel. Lishui displays the greatest imbalance across all resource types, with Gini coefficients of 0.461 (physicians), 0.507 (pharma-cists), 0.476 (beds), and 0.766 (equipment). This confirms that Lishui's high average accessibility scores coincide with severe internal inequity. The extreme values, especially for equipment, directly result from the aforementioned concentration of advanced, high-value resources in one or two major hospitals, leaving vast surrounding rural and mountainous areas with minimal access. This pattern highlights a critical limitation of relying solely on average SA metrics, as they can obscure profound localized shortages. In contrast, Huzhou achieves the most equitable allocation of physicians (Gini = 0.232), Shaoxing leads in pharmacist equity (Gini = 0.253), and Jiaxing ranks highest for both bed (Gini = 0.228) and equipment (Gini = 0.227) distribution.

**Table 3:** Equity assessment of spatial of different TCM healthcare resources at the provincial and municipal levels.

**Region**	**TCM Physician**	**TCM pharmacist**	**Bed**	TCM medical equipment^*^
Hangzhou	0.343	0.319	0.315	0.339
Huzhou	0.232	0.297	0.270	0.232
Jiaxing	0.246	0.254	0.228	0.227
Jinhua	0.311	0.285	0.325	0.462
Lishui	0.461	0.507	0.476	0.766
Ningbo	0.335	0.289	0.449	0.502
Quzhou	0.309	0.326	0.367	0.329
Shaoxing	0.266	0.253	0.282	0.264
Taizhou	0.339	0.320	0.340	0.406
Wenzhou	0.361	0.380	0.367	0.470
Zhoushan	0.444	0.383	0.336	0.385
The entire province	0.382	0.365	0.400	0.505

^*^TCM medical equipment worth more than 5,000 RMB.

**Figure 4:**
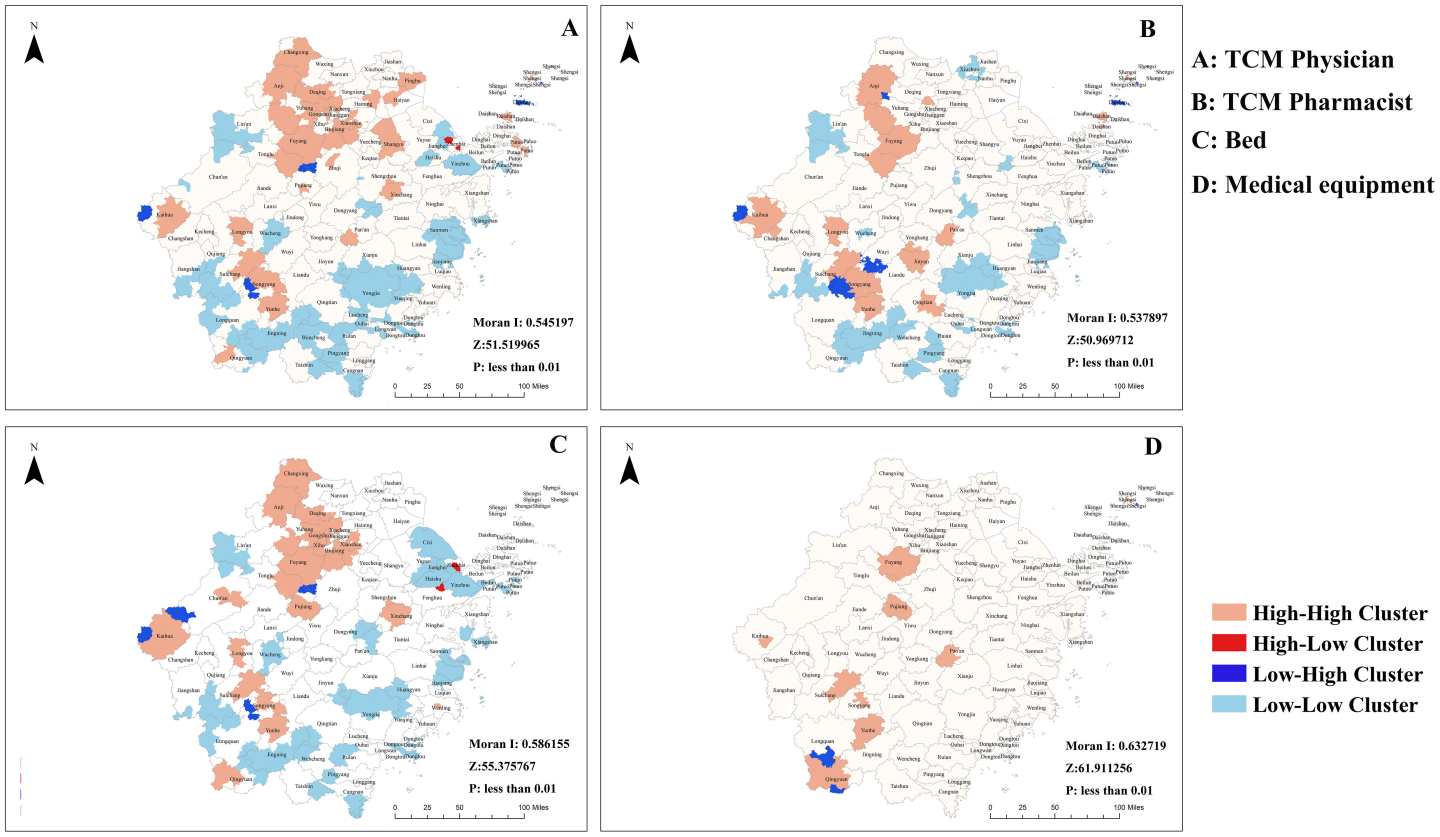
Cluster analysis of core resources spatial accessibility. **(A)** TCM physician; **(B)** TCM pharmacist; **(C)** bed; **(D)** TCM medical equipment.

Global Moran's *I* coefficients for all four resource indicators ranged from 0.598 to 0.708 (*p* < 0.05), confirming significant positive spatial autocorrelation across Zhejiang Province. Physician SA formed high–high clusters in the northern and southwestern regions and low–low clusters along the southern and eastern coasts (Figure 4A). Pharmacist SA also displayed high–high clustering in Hangzhou, Huzhou, Quzhou, and Lishui, with low–low clusters concentrated in the mountainous areas of Hangzhou, Jiaxing, Wenzhou, Taizhou, and Lishui (Figure 4B). Bed SA was similarly clustered, with pronounced high–high pockets around Hangzhou's urban core and southwestern Zhejiang, and low–low clusters in parts of Ningbo, Taizhou, Wenzhou, and Lishui (Figure 4C). Finally, medical equipment access exhibited high–high clusters in central and southwestern Zhejiang, alongside isolated low–high anomalies in sections of Lishui (Figure 4D). The spatial clustering patterns strongly correspond with regional socioeconomic gradients and topographic features. High-high clusters consistently align with economically developed plains and urban agglomerations, which benefit from cumulative resource investment and dense transport networks. Conversely, low-low clusters are predominantly located in economically less developed, mountainous, or peripheral coastal islands, where geographic barriers and lower development priority constrain both resource availability and travel connectivity. This persistent spatial heterogeneity underscores that accessibility is not merely a function of supply distribution but is also deeply mediated by underlying regional disparities.

## 4 Discussion

In China, TCM hospitals constitute the principal repository of high-quality TCM resources, and enhancing both their spatial accessibility and equity is fundamental to achieving the objectives of the “Healthy China Strategy” (28). The present study represents the first attempt to leverage navigation-derived travel-time estimates to quantify spatial accessibility to TCM hospitals in a designated pilot province, as well as to evaluate the equity of that accessibility across a comprehensive set of residential demand locations. Our analysis yielded three principal findings. First, 93.47% of village and community demand points and 97.21% of the provincial population are able to reach at least one sampled TCM hospital within a 1-h travel threshold, yet pronounced intercity variability remains. Second, Hangzhou and Lishui exhibit markedly superior accessibility across all four key resource indicators (TCM physicians, pharmacists, beds, and high-value equipment). Third, higher average SA does not guarantee equitable distribution: in Lishui, the measures of accessibility equity for all four resource categories meet or exceed thresholds indicative of significant disparity.

Our study advances the methodological assessment of healthcare accessibility by integrating high-resolution, dynamic travel time estimates into the 2SFCA method applied to TCM hospitals, a sector that has received limited attention. The 2SFCA method and its extensions (including the Enhanced 2SFCA and Gaussian 2SFCA) is widely applied to estimate SA to healthcare resources, serving as a proxy for real-world access to care (21, 29, 30). Crucial to the accuracy of 2SFCA analyses is the precision of travel-time estimates. In this study, we leveraged the real-time navigation API of Amap, which incorporates dynamic traffic factors (such as signal timings, vehicle flows, speed limits, and road conditions) to model residents' travel behavior more realistically than traditional network-analysis approaches (15, 24, 31). When combined with fine-grained, population-anchored demand points at the village and community committee level, the framework yields a more accurate and policy-relevant measurement of potential spatial access. Comparable navigation-based methods have been successfully validated in assessments of accessibility to primary healthcare services (18, 27). The proposed methodology is particularly well suited to the Chinese context, where rapid urbanization and dense traffic substantially affect travel costs, and it can be adapted to assess other specialty healthcare services with distinct utilization patterns.

Our analysis indicates that 93.47% of residential demand points and 97.21% of the provincial population can reach at least one sampled TCM hospital within a 1-h driving threshold. County-level hospitals contribute most substantially to this coverage, owing to their greater numbers and wider geographic dispersion. By comparison, only 68.8% of residential points in Shaanxi Province lay within 1 h of a county-level hospital (14), and approximately 90% of Nanjing residents could access general hospitals within the same threshold (32). These contrasts highlight Zhejiang's relatively high public TCM hospital coverage, which likely reflects its well-developed transportation network and more balanced allocation of resources. Moreover, the share of the population covered consistently exceeds the share of residential points covered, suggesting that areas with poor accessibility are predominantly remote and sparsely populated.

Our study reveals that the observed spatial patterns of accessibility and inequity are not merely a product of resource allocation but arise from a complex interplay between supply-side determinants and distinct demand-side behaviors within the TCM service system. The pronounced inequity in medical equipment distribution, far exceeding that of human resources, is a critical manifestation of this dynamic. On the supply side, high-value, capital-intensive equipment is predominantly concentrated in leading tertiary or municipal-level hospitals. This clustering results from economies of scale, historical investment patterns, and the broader socioeconomic gradient wherein wealthier regions possess greater fiscal capacity for advanced healthcare infrastructure (33). Consequently, this creates “islands of high capability”, as seen in Lishui, where excellent average accessibility masks severe local shortages in surrounding areas.

On the demand side, utilization patterns of traditional Chinese medicine distinctly shape patients' travel behavior. TCM services are often sought for chronic disease management and preventive care, contexts in which patients may demonstrate a greater willingness to travel in order to obtain perceived higher-quality care or specific therapeutic modalities. As a result, patients frequently bypass under-resourced local providers in favor of better-equipped central hospitals, thereby reinforcing existing spatial imbalances (33). However, effective access is strongly moderated by patient mobility. Older adults, who constitute a major group of TCM users (5), effectively face longer travel times because of functional limitations, rendering distance a substantial barrier to care (34). From a structural perspective, the organization of the TCM service system concentrates advanced resources within tertiary hospitals, which necessitates inter-regional travel for specialized services and further amplifies disparities between urban agglomerations and peripheral or geographically constrained areas.

The findings provide direct, actionable insights for Zhejiang in fulfilling its dual mandate as a national pilot for TCM reform and a demonstration zone for common prosperity. These policy frameworks collectively emphasize the establishment of a TCM service system with comprehensive urban–rural coverage, the equalization of basic public services, and the reduction of regional disparities in TCM development. Our results indicate that achieving these goals requires moving beyond aggregate coverage indicators toward interventions targeting the specific mechanisms underlying spatial inequity. For high-value equipment, regional equipment-sharing consortia among county-level hospitals or the deployment of mobile TCM diagnostic units could extend the functional reach of centralized resources to clustered rural townships. For human resources, sustained incentives for TCM practitioners to serve low-access cold-spot areas remain essential. More broadly, incorporating spatial accessibility metrics into the monitoring and evaluation of provincial TCM development plans would support more strategic infrastructure investment and contribute to narrowing persistent geographic gaps in access.

The study has several limitations. First, its cross-sectional design captures accessibility and resource distribution at a single time point, precluding assessment of temporal trends. Second, we analyzed only public TCM hospitals; exclusion of private clinics and community-level providers may give a partial view of the service landscape. Third, navigation API–derived travel times improve realism but carry inherent uncertainty (e.g., temporal sampling, routing heuristics, and rate-limit induced imputations), which can affect OD estimates. Fourth, spatial uncertainty remains: MAUP, positional errors in geocoding, and population-raster aggregation can influence accessibility metrics. Fifth, the 2SFCA model makes simplifying assumptions about facility choice and homogeneous demand within catchments, limiting behavioral realism. Finally, we did not incorporate socio-demographic need indicators (age, chronic-disease burden), which may modulate true service demand. These limitations do not invalidate our findings but indicate areas for future refinement.

## 5 Conclusion

This study highlights the complexities of SA to healthcare resources in TCM hospitals across Zhejiang Province. While the majority of residents can reach sampled hospitals within 1-h, significant disparities in SA to the four resource indicators exist across municipalities. Equity analyses further reveal concerning inequities in certain regions, with the most pronounced issues observed in the SA of TCM medical equipment resources. Methodologically, this study contributes to the literature by integrating real-time travel data and equity metrics to provide a refined assessment of TCM accessibility. Nevertheless, limitations related to cross-sectional design and model assumptions warrant further investigation using longitudinal data and expanded provider scopes. The findings offer actionable evidence for policymakers to support targeted resource allocation and advance equity-oriented TCM reforms.

## Data Availability

The original contributions presented in the study are included in the article/Supplementary material, further inquiries can be directed to the corresponding authors.
