# Supplementary material for: Spatial accessibility and equity of traditional Chinese medicine hospitals in Eastern China: application of improved two-step floating catchment area method
Source: Front Public Health. 2026 Feb 5;14:1741264. doi: 10.3389/fpubh.2026.1741264 (PMC12916626; doi:10.3389/fpubh.2026.1741264)
Supplement: Supplementary file 1 [file Table_1.docx]

Table S1. Distribution of TCM health resources in public TCM hospitals in different municipalities in Zhejiang Province

| Regional name | TCM physician | |  | TCM pharmacist | |  | Bed | |  | TCM device^*^ | |
| --- | --- | --- | --- | --- | --- | --- | --- | --- | --- | --- | --- |
|  | n (%) | Number per thousand people |  | n (%) | Number per thousand people |  | n (%) | Number per thousand people |  | n (%) | Number per thousand people |
| Hangzhou | 3148(32.5) | 0.258 |  | 535(25.6) | 0.044 |  | 14166(34.5) | 1.161 |  | 2875(31.7) | 0.236 |
| Huzhou | 532(5.5) | 0.156 |  | 107(5.1) | 0.031 |  | 2332(5.7) | 0.684 |  | 401(4.4) | 0.118 |
| Jiaxing | 734(7.6) | 0.133 |  | 122(5.8) | 0.022 |  | 2826(6.9) | 0.512 |  | 726(8.0) | 0.132 |
| Jinhua | 886(9.1) | 0.124 |  | 242(11.6) | 0.034 |  | 3834(9.3) | 0.538 |  | 1016(11.2) | 0.143 |
| Lishui | 384(4.0) | 0.153 |  | 116(5.6) | 0.046 |  | 1639(4) | 0.652 |  | 690(7.6) | 0.274 |
| Ningbo | 991(10.2) | 0.104 |  | 289(13.8) | 0.030 |  | 2805(6.8) | 0.294 |  | 549(6.1) | 0.058 |
| Quzhou | 346(3.6) | 0.151 |  | 83(4.0) | 0.036 |  | 1691(4.1) | 0.739 |  | 367(4.0) | 0.160 |
| Shaoxing | 877(9.0) | 0.164 |  | 141(6.7) | 0.026 |  | 3332(8.1) | 0.624 |  | 496(5.5) | 0.093 |
| Taizhou | 693(7.1) | 0.104 |  | 171(8.2) | 0.026 |  | 3385(8.2) | 0.508 |  | 968(10.7) | 0.145 |
| Wenzhou | 899(9.3) | 0.093 |  | 234(11.2) | 0.024 |  | 4508(11.0) | 0.467 |  | 789(8.7) | 0.082 |
| Zhoushan | 208(2.1) | 0.179 |  | 49(2.3) | 0.042 |  | 542(1.3) | 0.465 |  | 193(2.1) | 0.166 |

^*^TCM device worth more than 5000RMB
